# Longitudinal analysis of epigenome-wide DNA methylation reveals novel loci associated with BMI change in East Asians

**DOI:** 10.1186/s13148-024-01679-x

**Published:** 2024-05-27

**Authors:** Wenran Li, Mingfeng Xia, Hailuan Zeng, Huandong Lin, Andrew E. Teschendorff, Xin Gao, Sijia Wang

**Affiliations:** 1 CAS Key Laboratory of Computational Biology, Shanghai Institute of Nutrition and Health, University of Chinese Academy of Sciences, Chinese Academy of Sciences, Shanghai, China; 2 Department of Endocrinology and Metabolism, Zhongshan Hospital and Fudan Institute for Metabolic Diseases, Fudan University, Shanghai, China; 3 Department of Endocrinology and Metabolism, Wusong Branch of Zhongshan Hospital, Fudan University, Shanghai, China; 4 Human Phenome Institute, Fudan University, Shanghai, China; 5 Taizhou Institute of Health Sciences, Fudan University, Taizhou, Jiangsu, China; 6 Center for Excellence in Animal Evolution and Genetics, Chinese Academy of Sciences, Kunming, China

**Keywords:** Obesity, DNA methylation, BMI change, EWAS, East Asian population

## Abstract

**Background:**

Obesity is a global public health concern linked to chronic diseases such as cardiovascular disease and type 2 diabetes (T2D). Emerging evidence suggests that epigenetic modifications, particularly DNA methylation, may contribute to obesity. However, the molecular mechanism underlying the longitudinal change of BMI has not been well-explored, especially in East Asian populations.

**Methods:**

This study performed a longitudinal epigenome-wide association analysis of DNA methylation to uncover novel loci associated with BMI change in 533 individuals across two Chinese cohorts with repeated DNA methylation and BMI measurements over four years.

**Results:**

We identified three novel CpG sites (cg14671384, cg25540824, and cg10848724) significantly associated with BMI change. Two of the identified CpG sites were located in regions previously associated with body shape and basal metabolic rate. Annotation of the top 20 BMI change-associated CpGs revealed strong connections to obesity and T2D. Notably, these CpGs exhibited active regulatory roles and located in genes with high expression in the liver and digestive tract, suggesting a potential regulatory pathway from genome to phenotypes of energy metabolism and absorption via DNA methylation. Cross-sectional and longitudinal EWAS comparisons indicated different mechanisms between CpGs related to BMI and BMI change.

**Conclusion:**

This study enhances our understanding of the epigenetic dynamics underlying BMI change and emphasizes the value of longitudinal analyses in deciphering the complex interplay between epigenetics and obesity.

**Supplementary Information:**

The online version contains supplementary material available at 10.1186/s13148-024-01679-x.

## Introduction

Obesity is a major public health issue worldwide and is associated with a range of chronic diseases, including type 2 diabetes (T2D), cardiovascular disease, and certain types of cancer [1, 2]. Elevated Body Mass Index (BMI), a common measure of obesity, increase the risk of insulin resistance and impaired glucose tolerance, both of which are precursors of T2D [3]. Besides, changes in BMI, especially significant weight gain, are associated with increased risk of hypertension, dyslipidemia, and atherosclerosis, leading to higher rates of heart attacks and strokes and poorer cardiovascular health outcomes [4]. Overall, obesity is critically important for health outcomes due to its profound impact on various physiological systems and the development of chronic diseases [5]. Therefore, understanding the mechanism of BMI changes and implementing effective strategies for obesity prevention and management are critical for reducing the burden of obesity-related diseases and improving population health.

While common genetic variations account for more than 20% of variation in obesity [6], there is increasing evidence that epigenetic modifications, such as DNA methylation, may also play a role in mediating the correlation between environmental factors and obesity [7, 8]. DNA methylation is a process by which a methyl group is added to a cytosine residue in a DNA molecule, which can alter gene expression without changing the DNA sequence [9]. Several studies have investigated the association between DNA methylation and BMI [10, 11]. Overall, through EWAS analysis, more than 5000 DNA methylation sites have been identified to be associated with obesity-related traits [12]. Moreover, several recent studies indicated that weight gain at different stage of life had distinct causes [13] and weight gain across middle adulthood was significantly associated with major health outcomes [14]. However, most of these studies have been cross-sectional, which limits the ability to identify the mechanism of the dynamic changes of BMI across the lifespan and fails to detect the epigenetic markers that affects the change of BMI in middle-aged and elderly people.

BMI change, rather than just BMI itself, can provide important insights into the underlying mechanism that may contribute to BMI. From the epigenome-wide study of BMI change, we can identify biomarkers or molecular pathways that are involved in the development of obesity and related metabolic disorders. Longitudinal studies, which measure DNA methylation at multiple time points, are better suited for the identification of changes in DNA methylation associated with changes in BMI. Previous longitudinal EWAS analysis for BMI change has been conducted in Americans, Australians and European population [15–18], but rarely in East Asians. Since the diets and lifestyle habits of East Asians are discrepant from those of American or European populations, a different epigenetic mechanism underlying the longitudinal change of BMI in East Asians is highly possible.

Given the emerging evidence suggesting the role of epigenetic modifications, particularly DNA methylation, in obesity, we hypothesize that longitudinal changes in DNA methylation patterns are associated with changes in BMI over time in East Asians. To identify novel epigenetic loci that contribute to BMI change and elucidate the molecular mechanisms underlying obesity development, we conducted a longitudinal EWAS analysis of BMI change among 533 individuals of two Chinese cohorts who had at least two measurements of BMI and DNA methylation taken over a period of 4 years. We identified three novel CpGs (cg14671384, cg25540824, and cg10848724) significantly associated with BMI change in East Asians. We annotated these CpGs, explored their functional relevance, and revealed their expression patterns in obesity-relevant tissues. Our results provided insights into the epigenetic mechanisms underlying obesity progression.

## Methods

### Study participants

This study analyzed two Chinese cohorts derived from a large Chinese population study, the Shanghai Changfeng Study, composing of 6,595 individuals consecutively enrolled from Shanghai Changfeng Community, Shanghai from June 2009 to December 2012 [19]. The inclusion criteria were age 45 years or older, and the exclusion criteria included refusal to participate in the study or refusal to sign an informed consent form. Individuals in the cohort 1 included 407 Han Chinese who were randomly sampled from the Shanghai Changfeng study. Samples in the cohort 2 included another 126 Han Chinese individuals with high risk of new-onset type 2 diabetes from Shanghai Changfeng study with no overlap with cohort 1. The blood sample for DNA methylation was collected after an overnight fast of at least 12 h at the same day when BMI was measured for each participant. The study was approved by the Research Ethics Committee of Zhongshan Hospital, Fudan University (No. 2008–119 and B2013-132). Written informed consent was obtained from each participant.

### Blood DNA methylation analysis

Genome-wide DNA methylation profiles were obtained using the Illumina Infinium MethylationEPIC BeadChips following the manufacturer guide and protocol for Infinium MethylationEPIC array. Samples were randomized for each slide, plate and the position on plate, based on covariates including age, sex, and BMI, to remove any potential bias on DNA methylation measurement from technically-induced variation or confounding. Five hundred nanogram of genomic DNA from each blood sample was bisulfite converted using the EZ DNA Methylation Kit. DNA-BeadChip hybridization and single base extension were performed using a Freedom EVO robot. BeadChips were subsequently imaged using the iScan Microarray Scanner (Illumina), and Illumina.idat files were then processed with a R package named ChAMP [20]. Probes on chromosome X and Y and SNP-related probes were removed. The SNP list comes from [21]. Beta values were calculated corresponding to the ratio of the methylated signal over the sum signal, and P values were derived by comparing the sum signal to that of the background distribution. Betas with P values above than 0.01 were set to NA. Probes with less than 3 beads in at least 5% of samples per probe were filtered out. After quality control, beta values were normalized using a method named Beta Mixture Quantile (BMIQ). Batch effects were then corrected using a R package named ComBat [22]. The derived beta values were used for further analysis.

### Calculation of cell proportion in blood

Since heterogeneity in the composition of blood leukocyte cell types can confound the relationships between DNA methylation and phenotypes, we estimated the cell type abundance from methylation data using a R package named EpiDISH [23, 24]. The percentages of seven different cell types (CD4 T cells, CD8 T cells, NK cells, B cells, monocytes, and neutrophil) were calculated by mapping the beta values of CpGs to the reference values according to the database provided by EpiDISH.

### Epigenome-wide association study

A linear regression model was fitted to capture the correlation between DNA methylation and age acceleration, accomplished with a R package limma [25]. Age, sex, interval years, and blood leukocytes fractions (B cells, CD4 + and CD8 + T cells, NK cells, monocytes and neutrophils) were considered as covariates in the regression model. For the longitudinal EWAS analysis, the formula can be denoted as,

$${\boldsymbol{\Delta }{\varvec{M}}}_{{\varvec{i}}}={\beta }_{0}+ {{\varvec{\beta}}}_{{\varvec{S}}}{\boldsymbol{\Delta }{\varvec{B}}{\varvec{M}}{\varvec{I}}}_{{\varvec{i}}}+{\beta }_{age}{age}_{i}+{\beta }_{ageing}{IntervalYears}_{i}+{\beta }_{sex}{sex}_{i}+ \gamma ({cell proportions)}_{i}$$

where ${\boldsymbol{\Delta }M}_{i}$ is the change of methylation for $i$th subject, ${\boldsymbol{\Delta }{\varvec{B}}{\varvec{M}}{\varvec{I}}}_{{\varvec{i}}}$ the continuous value of BMI change for $i$th subject at baseline, ${age}_{i}$ and ${sex}_{i}$ the age and sex of $i$th subject, and $({cell proportions)}_{i}$ includes the predicted percentages of B cells, CD4 + and CD8 + T cells, NK cells, monocytes and neutrophils. Beside of the baseline model which considered age, sex, interval years, and cell fractions as covariates, we also built a secondary model which additionally adjusted for smoking and drinking status and achieved consistent results (Supplementary Text and Supplementary Fig. 1).

We also wondered whether the CpG sites associated with BMI change were also significantly associated with the cross-sectional BMIs. Therefore, we conducted cross-sectional EWAS of BMI to investigate the relationship between DNA methylation patterns and BMI levels at a specific point in time. The cross-sectional EWAS was separately performed in the baseline and follow-up data of cohort 1, adjusting for sex, age, and cell proportions.

### Meta-analysis of two cohorts

The meta-analysis was conducted using the tool METAL [26], which combine test statistics and standard errors across studies, taking sample size and direction of effect into account. In the meta-analysis, for each marker, a reference allele was selected and a z-statistic characterizing the evidence for association was calculated. The z-statistic summarized the magnitude and the direction of effect relative to the reference allele and all studies were aligned to the same reference allele. Next, an overall z-statistic and p-value were then calculated from a weighted sum of the individual statistics. Weights were proportional to the square-root of the number of individuals examined in each sample and selected such that the squared weights sum to 1.0. The process can be formulated as,

$$\widehat{\eta }= \sum_{k=1}^{K}{w}_{k} {\widehat{\eta }}_{k}$$

$${\text{Var}}\left(\widehat{\eta }\right)= \sum_{k=1}^{K}{w}_{k}^{2}{V}_{k}$$

where ${\widehat{\eta }}_{k}$ is the z-score from the *k*th study ($k\in \{\mathrm{1,2}\}$), ${V}_{k}$ the corresponding estimated variance, ${w}_{k}$ the weight used for the *k*th study in the meta-analysis.

The heterogeneity score was used to assess the degree of variability in effect sizes across the two cohorts. In the meta-analysis, we calculated the heterogeneity score, *I*-squared (*I*^2^), to represent the proportion of total variation in effect sizes that is due to between-study heterogeneity rather than sampling error. The formula can be denoted as,

$${I}^{2}=\frac{Q-df}{Q},$$

$$Q={\sum }_{k=1}^{K}({w}_{k}{({y}_{k}-\overline{y })}^{2})$$

where ${w}_{k}$ is the weight used for the *k*th study ($k\in \{\mathrm{1,2}\}$), ${y}_{k}$ the effect size in the *k*th study, $\overline{y }$ the average effect of all studies, $df$ the degree of freedom.

### Phenome-wide association analysis

Phenome-wide association study (PheWAS) analysis was applied to investigate the association between genetic variants and a wide range of phenotypic traits or conditions. Unlike traditional genome-wide association studies (GWAS), which typically focus on the association between genetic variants and a single phenotype, PheWAS simultaneously examines multiple phenotypes or traits. For each gene where the identified CpGs located, we performed the PheWAS analysis using the tool PhenoScanner [27], which searched for known associations between genetic variants within the given gene and various phenotypes by querying GWAS databases.

### Functional enrichment analysis

The power of a EWAS study much depends on the number of subjects provided by the cohort. Since our cohorts only contained 533 subjects, we loosened the threshold to *P* < 1 × 10^–5^ and selected the top 20 significant CpGs for further functional analysis. We annotated the detected CpGs to genes using R package *IlluminaHumanMethy–lationEPICanno.ilm10b4.hg19*. The gene ontology (GO) enrichment was conducted using the tool MetaScape [28]. An independent pathway enrichment in Reactome [29] and Kyoto Encyclopedia of Genes and Genomes (KEGG) pathways [30] was conducted using the R package clusterProfiler [31]. For each functional term or pathway to be analyzed, the Fisher’s test was performed to evaluate the enrichment. Specifically, we considered the number of total genes as *N*, the number of genes related to a specific function as *M*, the number of marker genes annotated from detected CpGs as *R*, and the overlap between marker genes and function-related genes as *k*. Then, the enrichment significance *p* value was calculated as the summation of the hypergeometric distribution, as.

$$p\mathrm{ value}\hspace{0.17em}=\hspace{0.17em}1-{\sum }_{i=0}^{k}\frac{\left(\begin{array}{c}M\\ i\end{array}\right)\left(\begin{array}{c}N-M\\ R-i\end{array}\right)}{\left(\begin{array}{c}N\\ R\end{array}\right)}$$

### Statistical analysis

All statistical analyses were performed using the R software version 3.6.2. The continuous parameters with normal distribution are presented as the means ± SD and skewed parameters are presented as the median with the interquartile range (25–75%) given in parentheses. All skewed parameters were normalized using rank-based inverse normal transformation before analysis. The continuous data with normal distribution were compared using the Student’s *t*-tests or one-way analysis of covariance (ANOVA), and the categorical variables using the chi-square test.

## Results

### Characteristics of the study population

Our study analyzed two population cohorts from Shanghai Changfeng Study, a community-based prospective cohort study of chronic diseases among middle-aged and elderly residents from Shanghai Changfeng Community. The average BMI levels of the participants from cohort 1 increased from 23.9 ± 3.0 kg/m^2^ to 24.7 ± 3.1 kg/m^2^ after an average of 4.1-year follow-up. In cohort 2, after an average of 4.4-year follow-up, the average BMI increased from 24.1 ± 2.5 kg/m^2^ to 24.5 ± 3.0 kg/m^2^. The percentage of smokers were lower in cohort 1 than that in cohort 2 (19% vs. 23%), while the percentage of drinkers were higher in cohort 1 than that in cohort 2 (18% vs. 9%). We further compared the BMI changes and global DNA methylation in smokers versus non-smokers, and drinkers versus non-drinkers. We found that there was no significant difference between smokers and non-smokers in terms of BMI change and global DNA methylation change in both cohorts (Supplementary Fig. 2A-B). Similar results were observed for drinkers and non-drinkers (Supplementary Fig. 2C-D). Detailed summary characteristics of the study participants were shown in Table 1.

**Table 1:** Study participant characteristics at baseline and follow-up of cohort 1 and cohort 2

	Cohort 1	Cohort 2
*Baseline*		
No. of participants	407	126
Male, n (%)	179 (44%)	66 (52%)
Age, years	61.7 ± 7.5	62.4 ± 8.6
BMI, kg/m^2^	23.9 ± 3.0	24.1 ± 2.5
Height, cm	162.1 ± 7.9	163.3 ± 8.7
Weight, kg	63.1 ± 10.0	64.5 ± 9.8
Cigarette smoking, n (%)	77 (19%)	29 (23%)
Alcohol drinking, n (%)	73 (18%)	11 (9%)
*Follow-up*		
Age, years	65.8 ± 7.4	66.7 ± 8.6
BMI, kg/m^2^	24.7 ± 3.1	24.5 ± 3.0
Height, cm	159.8 ± 8.2	163.3 ± 8.7
Weight, kg	63.1 ± 10.1	64.5 ± 9.8
*BMI change*		
Delta BMI, kg/m^2^	0.76 ± 1.4	0.40 ± 1.9
BMI change degree, kg/m^2^	0.03 ± 0.07	0.02 ± 0.08

### Epigenome-wide association analysis of BMI change

**Fig. 1:**
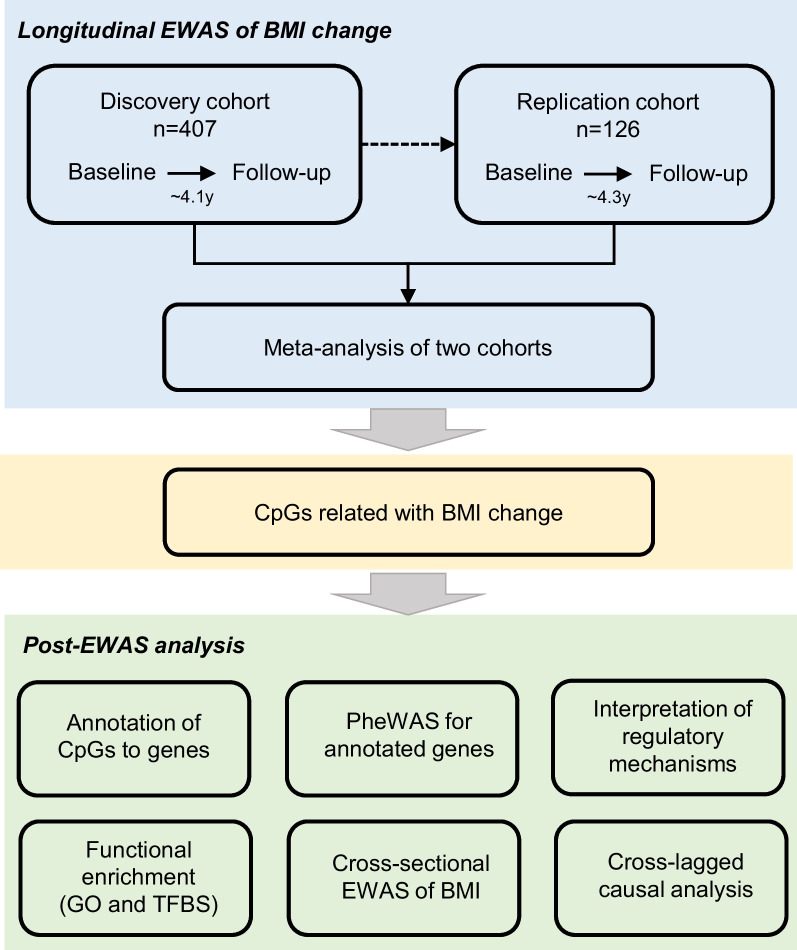
Study design

**Fig. 2:**
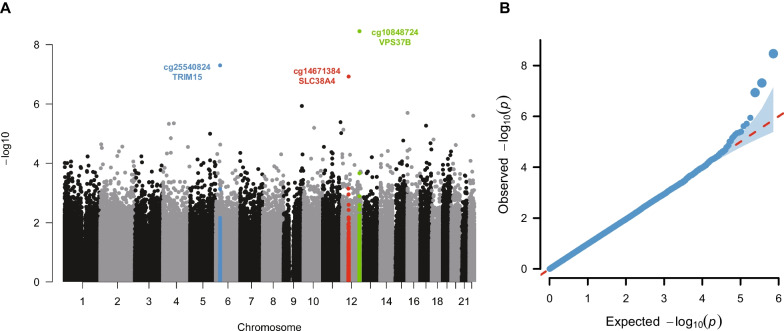
Meta-analysis results. **A** Manhattan plot showing the distribution of *P* values of the association of methylation probes with BMI change in the discovery cohort. The green dots indicate CpGs that fall within *VPS37B*; the blue dots indicate CpGs that fall within *TRIM15*; and the red dots indicate CpGs that fall within *SLC38A4*. **B** Q-Q plot showing enrichment *P* values of the discovery cohort compared against those of randomly selected baseline samples

**Fig. 3:**
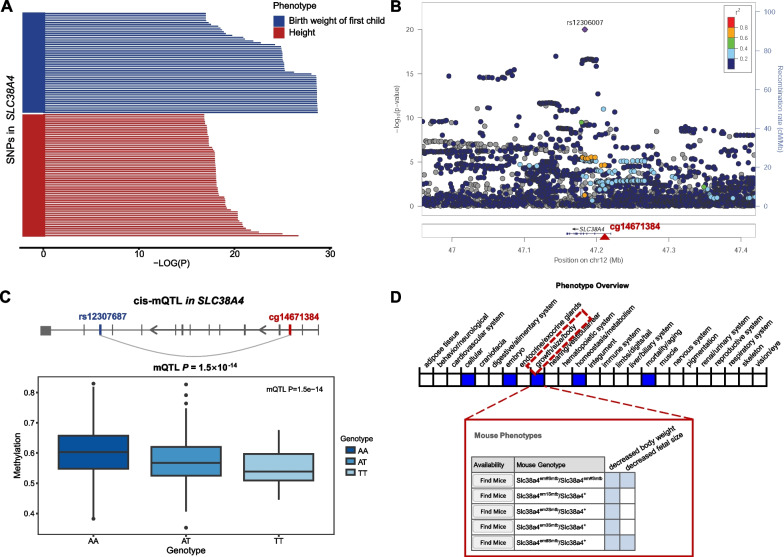
Interpretation for *SLC38A4*. **A** PheWAS results for *SLC38A4*. Blue bars represent GWAS significance of phenotypes related to birth weight; red bars represent those related to height. **B** Zoom locus of *SLC38A4* in the GWAS of body height. The red triangle shows the position of cg14671384. **C** A cis-mQTL association between the GWAS signal (rs12307687) and the EWAS signal (cg14671384). **D** The influence of *SLC38A4* mutations on mouse phenotypes. Highlighted blue boxes represent the observed phenotype change

We used the BMI change rate over baseline to describe BMI change and conducted longitudinal EWAS analysis on BMI change in cohort 1 and cohort 2, where the EWAS model was described in Methods and Supplementary Text. The overall framework of this study was summarized in Fig. 1. We regarded cohort 1 as the discovery cohort and cohort 2 as the replication cohort. We identified three CpG sites significantly associated with BMI change in the discovery cohort (*P* < 1 × 10^–6^) and all of the three CpGs showed the same effect directions in the replication cohort. Then, we performed a meta-analysis using the tool METAL [26] to integrate the EWAS results of cohort 1 and cohort 2 (Supplementary Table 1). The meta-analysis further confirmed the three CpG sites significantly associated with BMI change in the discovery cohort (Fig. 2 and Table 2). The most relevant CpG site (cg14671384, *P* = 9.09 × 10^–9^) locates in the promoter of the *SLC38A4* gene (Solute Carrier Family 38 Member 4), which has been reported to be a transporter of cationic and neutral amino acids and closely related with glucagon and hyperglycaemia [32, 33]. To systematically investigate the function of *SLC38A4*, we performed the PheWAS analysis using the tool PhenoScanner [27] (Fig. 3A). We found that there were significant GWAS signals in *SLC38A4* for phenotypes of birth body weight (e.g. rs180438, *P* = 9 × 10^–21^ in GCST008363) [34] and body height (e.g. rs12306007, *P* = 1 × 10^–20^ in GCST90018959; Fig. 3B) [35, 36], indicating the possible participation of *SLC38A4* in the formation of body shape. More interestingly, we observed a significant association (*P*_mQTL_ = 1.5 × 10^–14^) between the CpG cg14671384 and a significant height-related SNP rs12307687 (GWAS *P* = 1 × 10^–8^) [37] in an Asian mQTL database named Pan-mQTL (Fig. 3C). Besides, a previous mouse study [38, 39] observed a decreased body weight in *SLC38A4* knock-out mice, supporting a direct role of *SLC38A4* in obesity (Fig. 3D).

**Table 2:** CpGs significantly associated with BMI change in the meta-analysis

CpG	CHR	Position	Gene	Discovery cohort			Replication cohort		Meta-analysis		
				Effect	*P* Value	FDR	Effect	*P* Value	Direction	*I*^2^	Summary *P* Value
cg14671384	12	47,219,920	*SLC38A4*	− 0.341	1.19E−07	2.82E−02	− 3.25E−03	6.48E−02	− −	0.34	9.09E−09
cg25540824	6	30,139,686	*TRIM15*	0.967	4.98E−08	1.77E−02	2.04E−03	5.82E−02	+ +	0.65	7.05E−08
cg10848724	12	123,380,878	*VPS37B*	1.065	3.52E−09	2.51E−03	2.88E−03	7.49E−02	+ +	0.52	5.85E−07

^*^I^2^ is the heterogeneity score that assess the degree of variability in effect sizes across the two cohorts

Another two significant CpG sites (cg25540824, *P* = 7.05 × 10^–8^; cg10848724, *P* = 5.85 × 10^–7^) were respectively annotated to a CpG island shore and a CpG island. Specifically, cg25540824 locates in the intron 7 of the *TRIM15* gene (Tripartite Motif Containing 15) and maps to the binding site of the well-known transcription factor CTCF, which is usually active in regulatory regions [40]. cg10848724 locates in the promoter of the *VPS37B* gene (Vacuolar Protein Sorting-Associated Protein 37B). We found significant GWAS signals in *VPS37B* for phenotypes of BMI-adjusted waist-hip ratio and BMI-adjusted hip circumference [41], demonstrating the close relationship between *VPS37B* and BMI-related phenotypes (Supplementary Fig. 3). Additionally, according to gene ontology (GO) database [42], *VPS37B* is involved in cell differentiation and enables calcium-dependent protein binding, which can influence the dynamic change of BMI [43].

### Interpretation of the regulatory mechanisms of the identified CpGs

**Fig. 4:**
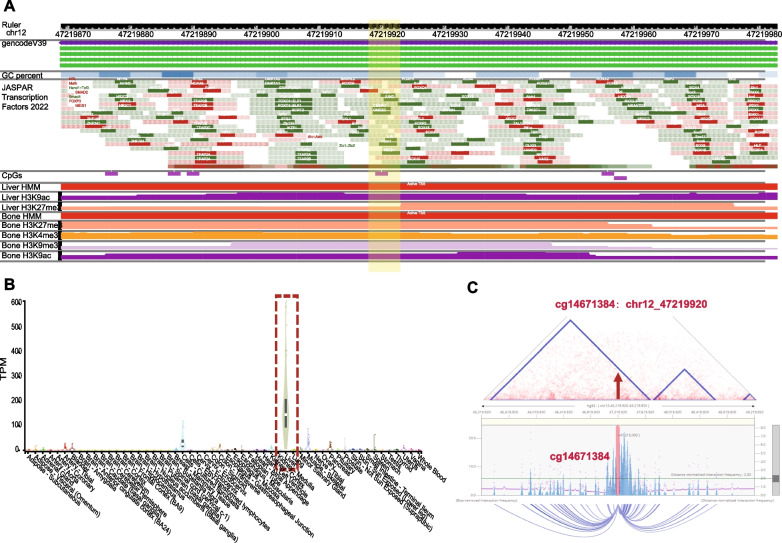
Regulatory activity of cg14671384. **A** The activity of the genomic region where cg14671384 locate. From top to bottom, the bars show the location, GC percent, Jaspar TFs, and different histone marks. The height in each bar represents the activity of the corresponding position. cg14671384 was shadowed in yellow. **B** The expression of *SLC38A4* in different tissues. **C** The 3D interactions in the genomic region where cg14671384 locate. cg14671384 was marked in red

To understand the regulatory mechanism of the identified CpGs, we first checked the transcription factors that bind to cg14671384 using the tool JASPAR [44], and observed the binding of TF *FOSL1*, *GMEB2*, and *AHR* on this CpG site, all of which were involved in RNA polymerase II-specific cis-regulation (Fig. 4A) [45]. Then, we checked the tissue-specific expression of *SLC38A4* (where cg14671384 is located) using GTEx [46] and observed that *SLC38A4* was significantly overexpressed in liver, which is a tissue highly relevant to obesity and the change of body weight (Fig. 4B) [47]. Furthermore, we found there were ChIP-seq signals of different chromatin states and histone marks at cg14671384 in liver (Fig. 4A), as well as dense Hi-C interactions between the locus of the identified CpG and distant regions (Fig. 4C), together implying the active regulatory role of cg14671384. Interestingly, we noticed that there was a significant eQTL (*P* = 1.70 × 10^–7^) located in an enhancer 100 kb away from *SLC38A4*, and a significant 3D interaction (*P* = 2.05 × 10^–5^) was captured between this gene and the enhancer [48] (Supplementary Fig. 4). Based on these observations, we inferred a possible regulatory process that the distal SNP changes the function of the enhancer where it locates, then the enhancer regulates the CpG through the chromatin 3D interaction and changes the expression of the downstream gene *SLC38A4*.

Next, we checked the activity of the other two CpG sites in the above regulatory signals. For cg10848724, we observed the binding of TF *ZFP57*, *RUNX1*, and *TFAP2E* on this CpG site (Supplementary Fig. 5A), of which *ZFP57* and *RUNX1* functions as transcriptional repressors and *TFAP2E* acts within positive regulation of transcription [45]. The gene *VPS37B* where cg10848724 locates was highly expressed in esophagus tissue (Supplementary Fig. 5B), which participated in the digestive process and may correlated with body weight [49]. For cg25540824, we observed the binding of TF *ZFP57*, *ELF2*, *MEIS1* and *NFIX* (Supplementary Fig. 6A), all of which were involved in the DNA-binding transcription repressor activity [45]. The corresponding gene *TRIM15* is significantly over-expressed in the colon, liver, and small intestine tissues (Supplementary Fig. 6B), which may relate with the change of BMI by influencing body weight. Similarly, we found there were ChIP-seq signals of different chromatin states and histone marks in the relevant tissues at both CpG sites and also dense Hi-C interactions from these CpGs to distant regions (Supplementary Fig. 5C and 6C), indicating that these CpG sites are actively involved in regulatory processes.

### Functional analysis of CpGs related to BMI change

We loosened the threshold for EWAS results to *P* < 1 × 10^–5^ and selected the top 20 significant CpGs for the functional analysis. We annotated those CpGs to coding regions of 13 genes and investigated the annotated genes using the tool EWAS atlas [12]. Results showed that 11 out of the 13 genes have been reported to have associations with obesity or T2D (Table 3). Then, we conducted functional enrichment analysis using MetaScape [28] and observed significant enrichment of those genes in negative regulation of protein phosphorylation (GO:0001933; Fisher exact test *P* = 2.84 × 10^–4^) and negative regulation of cell migration (GO:0030336; Fisher exact test *P* = 3.65 × 10^–4^; Supplementary Fig. 7A). Protein phosphorylation and cell migration can both be induced by growth factor [50–52] and plays important roles in the development of body height and obesity [53, 54].

**Table 3:** Annotation for the top 20 CpGs that most significantly associated with BMI change. The related traits were phenotypes reported to be significantly correlated with the gene where the identified CpGs located, collected from EWAS atlas

CpG	CHR	Position	Relation to Island	Gene	Reported trait in EWAS atlas
cg10848724	12	123,380,878	Island	*VPS37B*	obesity [55]; type 2 diabetes (T2D) [56]
cg25540824	6	30,139,686	N_Shore	*TRIM15*	obesity [55]; type 2 diabetes (T2D) [56]
cg14671384	12	47,219,920	OpenSea	*SLC38A4*	birth weight [57]; weight loss [58]
cg16439649	9	129,175,984	OpenSea	*MVB12B*	type 2 diabetes (T2D) [56]
cg07312485	16	2,052,423	Island	*ZNF598*	obesity [55]; type 2 diabetes (T2D) [56]
cg19210553	22	44,438,952	OpenSea	*PARVB*	obesity [55]
cg14575164	11	124,951,616	OpenSea	*SLC37A2*	differentiation of skeletal muscle [59]
cg03578005	4	77,170,672	N_Shore	*\*	\
cg23433430	4	41,362,520	Island	*LIMCH1*	obesity [55]; type 2 diabetes (T2D) [56]
cg03864245	17	41,855,745	Island	*DUSP3*	multiple sclerosis [60]
cg08388111	10	74,032,820	N_Shore	*DDIT4*	obesity [61]
cg24085975	12	10,657,644	OpenSea	*EIF2S3L*	infant sex [62]; gender [63]
cg16282618	11	129,444,501	OpenSea	*\*	psoriasis [64]
cg02539153	5	140,614,765	N_Shore	*PCDHB18; CH17-40K24.2*	obesity [61]; type 1 diabetes (T1D) [65]
cg10742523	4	55,650,393	OpenSea	*\*	obesity
cg21349637	18	13,218,607	Island	*C18orf1; LDLRAD4*	obesity [55]; type 2 diabetes (T2D) [56]
cg21850735	15	73,919,896	OpenSea	*NPTN*	obesity [61]
cg13093727	17	72,767,907	OpenSea	*NAT9*	\
cg17021949	2	6,589,760	OpenSea	*\*	ancestry [66]
cg13806964	6	31,165,914	Island	*HCG27*	obesity [55]; type 2 diabetes (T2D) [56]

Besides, we investigated the enrichment significance of the annotated genes in Reactome [29] and Kyoto Encyclopedia of Genes and Genomes (KEGG) pathways [30] using the R package clusterProfiler [31]. Results showed that the annotated genes were mainly enriched in basic cell signaling processes like membrane binding and targeting of glycosaminoglycan (GAG) proteins (Fisher exact test *P* = 4.23 × 10^–5^; Supplementary Fig. 7C) and endocytosis (Fisher exact test *P* = 7.83 × 10^–3^; Supplementary Fig. 7D).

Next, we further checked the enrichment of transcription factor binding sites (TFBS) on the selected CpGs and observed a significant enrichment of the CpGs in the binding of TF *FOXE1* (Fisher exact test *P* = 2.51 × 10^–3^; Supplementary Fig. 7B). *FOXE1* is a well-known thyroid-marker protein that plays an important role in cell growth and migration [67, 68], while thyroid mainly regulates the growth rate, promotes the metabolism, and maintains the growth and development of the body [69, 70]. Moreover, *FOXE1* has been reported to be associated with the abnormality of body height by Human Phenotype Ontology (HP:0000002) [71], indicating a possible regulatory process from the TF binding of detected CpGs to body height change. Taken together, the enrichment analysis demonstrated that CpGs detected by the longitudinal EWAS of BMI change were valuable and might relate to the development of body height and obesity.

### Sex stratification analysis

We further conducted stratified EWAS analyses for males and females to investigate how DNA methylation patterns are associated with BMI change within different sex group. There were two CpGs (cg25540824 and cg10848724) detected for males in cohort 1 at the threshold of *P* < 1 × 10^–6^, while no signal was identified for females (Supplementary Fig. 8A-B). Then, we systematically compared the effect size of CpGs in males with that in females, and found significant inconsistency across the two genders (Pearson’s correlation coefficient = 0.52; Supplementary Fig. 8C), indicating a possible difference on the sex-specific epigenetic regulation of BMI change and obesity development.

### Comparison with the cross-sectional epigenome-wide associations of BMI

We wondered whether the CpG sites associated with BMI change were also significantly associated with the cross-sectional BMIs. To this end, we separately conducted cross-sectional EWAS analysis using the baseline and follow-up data of cohort 1. Then, we collected the published BMI-related CpGs from previous studies (Supplementary Table 2) [10, 16, 17] and checked the significance of the published CpGs in the cross-sectional EWAS of BMI and the longitudinal EWAS of BMI change. As expected, the published BMI-related CpGs were much more significant than random CpGs in EWAS results of BMI (baseline: *t*-test *P* = 6.30 × 10^–7^; follow-up: *t*-test *P* = 2.72 × 10^–11^; Supplementary Fig. 9A-F), but did not show significant difference with random CpGs in EWAS results of BMI change (*t*-test *P* = 0.45; Supplementary Fig. 9G-I). This suggests BMI and BMI change may have different regulatory mechanisms involving distinguished CpGs. Besides, we checked the correlation between cross-sectional BMI and the methylations of the CpG identified to be correlated with BMI change, and observed that the CpGs significantly associated with BMI change were not correlated with either baseline or follow-up BMI (Supplementary Fig. 10).

## Discussion

In this study, we conducted a longitudinal association analysis between epigenome-wide DNA methylation and BMI change in an East Asian population. The longitudinal EWAS analysis identified three novel loci associated with BMI change, two of which were located in genes that have previously been related to body shape, height, weight, or basal metabolic rate. We found 11 out of the 13 genes where the top 20 CpGs located were previously reported to be related with obesity or T2D. Besides, functional enrichment analysis revealed that the top CpGs localized to genes involved in functions related to protein phosphorylation and cell migration, which can both be induced by growth factor, while growth factor plays important roles in the development of body height and obesity. These findings provide comprehensive evidence for the role of the identified CpGs in the change of BMI.

The phenotype-wide association analysis demonstrated that the identified CpG sites were located in regions associated with basal metabolic rate and a range of body shape-related traits, including height, weight, and body fat mass. This suggests the changes in DNA methylation at the identified loci may have broader effects on body shape-related phenotypes. Overall, the results of PheWAS analysis showed that the identified CpG sites were in accordance with previous findings from GWAS of BMI, revealing that the EWAS of BMI change and the GWAS of BMI are relevant and the epigenetic changes may contribute to the missing heritability of obesity.

The epigenomic data of TF motif, chromatin state and histone modification were generated by various ChIP-seq experiments which provided valuable insights into the functional regulation of genomic regions such as CpGs. For example, TF motif analysis allowed us to identify transcription factor binding motifs enriched in the vicinity of the identified CpGs, providing clues about potential transcriptional regulatory mechanisms underlying BMI change [72]. Chromatin state data reflected the structural and functional organization of chromatin, providing insights into the regulatory elements and functional states associated with the identified CpGs [73]. Histone modification profiling enabled us to assess the epigenetic modifications associated with active or silenced chromatin states, further elucidating the regulatory potential of the CpGs. Given the interactive nature of epigenomic processes, we integrated these diverse epigenomic datasets to comprehensively investigate the regulatory activity of the identified CpGs. By examining the concordance or discordance between DNA methylation patterns and other epigenomic features, we gained insights into the coordinated regulation of the identified CpGs and cellular phenotypes associated with BMI change.

Besides, we performed the cross-sectional EWAS of BMI, respectively in baseline and follow-up. Surprisingly, the CpGs identified in the longitudinal EWAS for BMI change were not significantly associated with baseline or follow-up BMI. This finding highlights the importance of studying BMI change specifically, as the underlying epigenetic mechanism of BMI change may not be the same as that of BMI itself. However, most of the previous researches typically focused on the study of BMI, taking BMI change as an identical phenotype of BMI [74]. There were also several studies that noticed the difference between BMI and BMI change and explored the epigenetic makers associated with BMI change [17]. Nevertheless, these studies of BMI change usually use the linear mixed model to identify significant CpG signals, which cannot capture direct information of BMI changing rate. We show that by using linear mixed model, we can only obtain similar results with the cross-sectional EWAS results of BMI (Supplementary Text and Supplementary Fig. 11). More importantly, the previous studies were performed in the American or European longitudinal cohorts, leaving the mechanism of BMI longitudinal change not well-explored in East Asian population (Supplementary Text). Therefore, in this study, we designed a statistic of BMI changing rate which is defined as the rate of BMI change from baseline to follow-up over the interval time, to focus specifically on epigenetic signals associated with the change of BMI over time. Thus, we can detect specific signals unique to BMI change. The present results suggest that studying BMI change as a distinct phenotype can provide valuable insights into the underlying epigenetic mechanisms involved in weight gain or loss over time.

However, this study also had several inevitable limitations. The major drawback of the present study was its limited sample size, where we only included 533 longitudinal samples for the meta-analysis. The sample scale of an EWAS study directly influences the number of identified CpGs, as well as their significance. Longitudinal analysis of BMI change should be conducted in larger datasets in the future. Second, the CpGs identified by the discovery cohort showed the same effect direction in the replication cohort, but the associations were not significant in the replication cohort. One possible reason for this might be that the replication cohort only contained 126 samples. Third, our study investigated the TF binding status, chromatin states, expression levels and physical interactions of the identified CpGs, to infer their possible role in the mechanism of BMI. This inference was only based on public sequencing data. Further biological experiments need to be conducted to explore and confirm the mechanism of how the identified CpGs influence the change of BMI. Fourth, it would be a possible improvement if the DNA methylation data can be generated in an obesity-related tissue. By conducting EWAS analysis using DNA methylation data generated in specific tissues, such as adipose tissue or liver, we can capture DNA methylation changes specifically relevant to the expression of genes involved in adipogenesis, lipid metabolism, insulin signaling, and other pathways relevant to BMI changes.

In conclusion, despite the limitations discussed above, this longitudinal study provides important insights into the complex interplay between the change of BMI and DNA methylation. The results of this study showed three CpGs significantly associated with BMI change, two of which located in regions that were previously reported to be related to body shape or basal metabolic rate, providing further evidence for the relevance of these loci in relation to BMI. Additionally, the study used various approaches such as GWAS, PheWAS, and functional enrichment analyses to further characterize these BMI change-associated CpGs. Furthermore, the study explored the CpGs in terms of their TF binding status, chromatin states, expressions of corresponding genes, and distant 3D interactions, revealing that all the detected CpGs are actively involved in regulation processes and are highly expressed in obesity-relevant tissues. This provides further evidence for the potential role of these CpGs in the development of obesity and related diseases. It is noteworthy that our study revealed differences between the CpGs identified by EWAS of BMI change and those identified by cross-sectional EWAS of BMI. This distinguished our analysis of BMI change with previous BMI EWAS analysis and highlighting the value of the longitudinal analysis in providing a more comprehensive understanding of the role of DNA methylation in BMI change and the need to set up future larger longitudinal EWAS studies. Overall, the identification of novel CpG sites associated with BMI change and their functional characterization provides a potential insight for the development of new biomarkers and therapeutic targets for obesity and related metabolic diseases.

### Supplementary Information

Additional file 1.

Additional file 2.

Additional file 3.

## Data Availability

The raw data files of two CF cohorts were deposited in https://www.biosino.org/download/node/data/public/OED874656 and https://www.biosino.org/download/node/data/public/OED874655. The summary statistics of EWAS analysis were available in the supplementary Table 1.
