# Supplementary material for: Longitudinal analysis of epigenome-wide DNA methylation reveals novel loci associated with BMI change in East Asians
Source: Clin Epigenetics. 2024 May 27;16:70. doi: 10.1186/s13148-024-01679-x (PMC11131215; doi:10.1186/s13148-024-01679-x)
Supplement: Supplementary file 1 — Additional file 1. [file 13148_2024_1679_MOESM1_ESM.pdf]

Longitudinal analysis of epigenome-wide DNA methylation reveals novel loci associated with BMI change in East Asians

Wenran Li[1,*], Mingfeng Xia[2,3*], Hailuan Zeng[2,4], Huandong Lin[2], Andrew E. Teschendorff[1], Xin Gao[2,4#], Sijia Wang[1,5,6,#]

[1]CAS Key Laboratory of Computational Biology, Shanghai Institute of Nutrition and Health, University of Chinese Academy of Sciences, Chinese Academy of Sciences, Shanghai, China.

[2]Department of Endocrinology and Metabolism, Zhongshan Hospital and Fudan Institute for Metabolic Diseases, Fudan University, Shanghai, China

[3]Department of Endocrinology and Metabolism, Wusong Branch of Zhongshan Hospital, Fudan University, Shanghai, China.

[4]Human Phenome Institute, Fudan University, Shanghai, China.

[5]Taizhou Institute of Health Sciences, Fudan University, Taizhou, Jiangsu, China.

[6]Center for Excellence in Animal Evolution and Genetics, Chinese Academy of Sciences, Kunming, China.

[*]These authors contributed equally.

[#]Corresponding authors:

Xin Gao, Email: zhongshan_endo@126.com, ORCID: 0000-0003-1864-7796.

Sijia Wang, Email: wangsijia@picb.ac.cn. ORCID: 0000-0001-6961-7867.

Supplementary Texts

The linear mixed model

The linear mixed model (LMM) is a powerful statistical tool used to analyze data collected over time from the same individuals or subjects, which can account for within-subject correlations and heterogeneity in the data, while also allowing for the estimation of fixed and random effects. In cohort 1, we conducted the LMM analysis using the "lme4" package in R [1], which specified the relationship between the outcome variable and the predictor variables, including fixed effects and random effects. The formula of LMM can be denoted as,

$$\boldsymbol{M_i} = (\beta_0 + u_{0i}) + (\beta_s + u_{1i})\boldsymbol{BMI_i} + \beta_{age} age_i + \beta_{sex} sex_i + \gamma(cell\,proportions)_i + \varepsilon_i,$$

where M_i is the methylation for i th subject, $\boldsymbol{BMI_i}$ the BMI for i th subject at baseline, age_i and sex_i the age and sex of i th subject, and $(cell\,proportions)_i$ includes the predicted percentages of B cells, CD4+ and CD8+ T cells, NK cells, monocytes and neutrophils. u_{0i} is the random intercept modelling baseline individual heterogeneity, u_{1i} the random slope modelling individual heterogeneity in the relationship, where both u_{0i} and u_{1i} are assumed Gaussian distribution.

We compared the EWAS results of cohort 1 using LLM with the cross-sectional results using traditional regression models, and observed similar EWAS results (Supplementary Figure 11). The Pearson's correlation coefficient (PCC) between EWAS results calculated using LMM and the EWAS results of baseline BMI was 0.84 (two-sided t-test $P = 3.37\times10^{-284}$), while the PCC between EWAS results using LMM and the EWAS results of follow-up BMI was 0.86 (two-sided t-test $P = 1.11\times10^{-212}$).

A secondary EWAS model adjusting for smoking and drinking

We built a secondary model to include smoking and drinking as confounders. The formula of the secondary model is as follows:

$$\boldsymbol{\Delta M_i} = \beta_0 + \boldsymbol{\beta_S \Delta BMI_i} + \beta_{age} age_i + \beta_{ageing} IntervalYears_i + \beta_{sex} sex_i$$

$$+ \beta_{drinking}\boldsymbol{Drinking_i} + \beta_{smoking}\boldsymbol{Smoking_i} + \gamma(cell\, proportions)_i,$$

where $\boldsymbol{\Delta M_i}$ is the change of methylation for i th subject, $\boldsymbol{\Delta BMI_i}$ the continuous value of BMI change for i th subject at baseline, age_i and sex_i the age and sex of i th subject, $Drinking_i$ and $Drinking_i$ the status of i th subject, and $(cell\, proportions)_i$ includes the predicted percentages of B cells, CD4+ and CD8+ T cells, NK cells, monocytes and neutrophils.

On the basis of the baseline model, the secondary model additionally adjusted for smoking and drinking status. The secondary model identified the same CpGs (cg14671384, cg25540824, and cg10848724) as the baseline model at the threshold of $P < 1\times10^{-6}$. We further compared the effect sizes in EWAS results of these two models. Results showed that the effect sizes of the secondary model adjusting for drinking and smoking was highly consistent with the effect size of our baseline model (Pearson's correlation coefficient = 0.997; Supplementary Figure 1), indicating that the EWAS of BMI change was not sensitive to smoking and drinking.

DMR analysis

We conducted the differential methylation region (DMR) analysis using the R package DMRcate [2], to detect genomic regions with differential DNA methylation patterns correlated with BMI change. First, we identified differential methylation positions (DMPs) using the "cpg.annotate" function of DMRcate. Then, DMRcate used a spatial kernel smoothing approach to model DNA methylation levels across the genome and conducted hypothesis tests to determine DMR regions. The identified DMRs were contiguous genomic regions with consistent methylation differences and were assigned with significance scores based on statistical testing results. We limited DMRs to contain at least three CpG signals to ensure the confidence of the identified regions, as in [3, 4].

Using the methods described above, we identified a DMR of "chr20:57427472-57427713" located near *GNAS*. *GNAS* contains a differentially methylated region (DMR) at the 5' exons, which is commonly found in imprinted genes and correlates with transcript expression. Because of imprinting, mutations on the maternal allele of *GNAS* can cause obesity and hormone resistance (pseudohypoparathyroidism) [5].

However, the CpGs identified by the longitudinal EWAS analysis did not fall into this DMR. DMR analysis can be considered as a supplementary to the DMP analysis.

Comparison of EWAS of BMI change in different populations

Demerath et al conducted a EWAS analysis of BMI change with 2097 African American adults in the Atherosclerosis Risk in Communities (ARIC) study as the discovery cohort and 2377 White adults in the Framingham Heart Study as the replication cohort [6]. In the study, 8 CpGs (cg15871086, cg09554443, cg26403843, cg07136133, cg13123009, cg00574958, cg03546163, and cg16672562) were identified to be significantly associated with BMI change. According to their analysis, the CpGs identified in American adults were mainly near genes involved in lipid metabolism, immune response/cytokine signaling and other diverse pathways. We compared the CpGs identified in American population with those identified in Asian population and found no overlap between the two studies. Besides, the top CpGs identified in Asian population in negative regulation of protein phosphorylation and cell migration, which can both be induced by growth factor and plays important roles in the development of body height and obesity [7, 8]. The difference between CpGs identified in different populations may reflect underlying genetic variation that is specific to each population. Differences in diet, physical activity, socioeconomic status, and other environmental factors may lead to population-specific associations between CpG sites and BMI change. Overall, differences in CpGs identified in different populations highlight the complex interplay between genetic, environmental, and epigenetic factors in shaping BMI-related phenotypes and underscore the importance of considering population-specific factors in epigenome-wide association studies.

Supplementary Figures

Supplementary Figure 1. Comparison between the effect sizes of the baseline model and those of the secondary model adjusting for drinking and smoking.

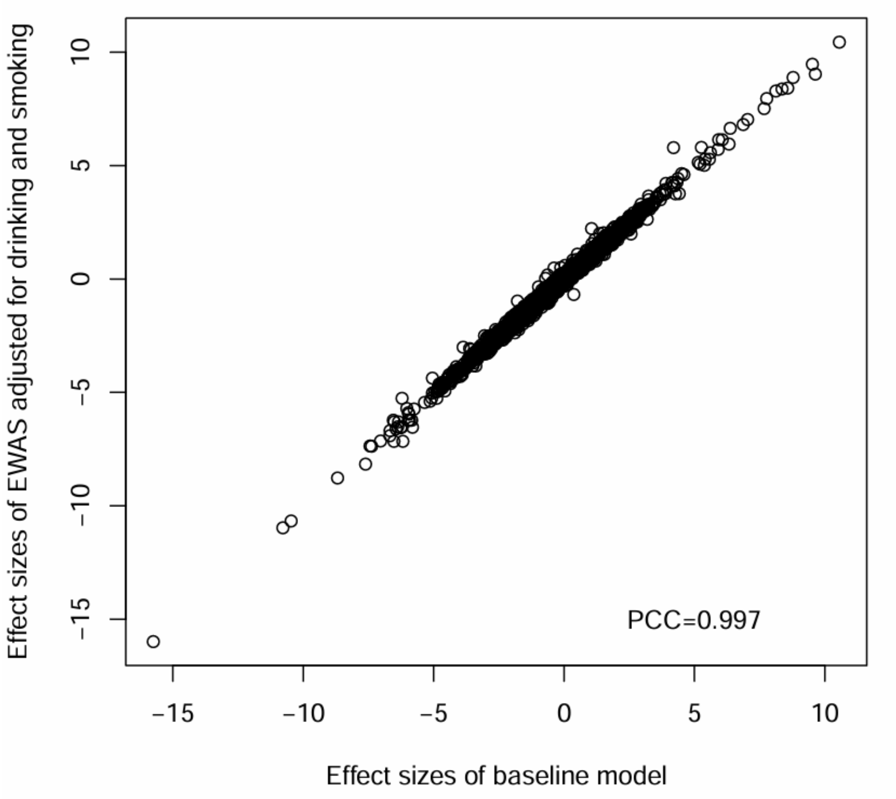

Supplementary Figure 2. Comparison between the BMI changes and global DNA methylation in smokers versus non-smokers (A, B), and drinkers versus non-drinkers (C, D).

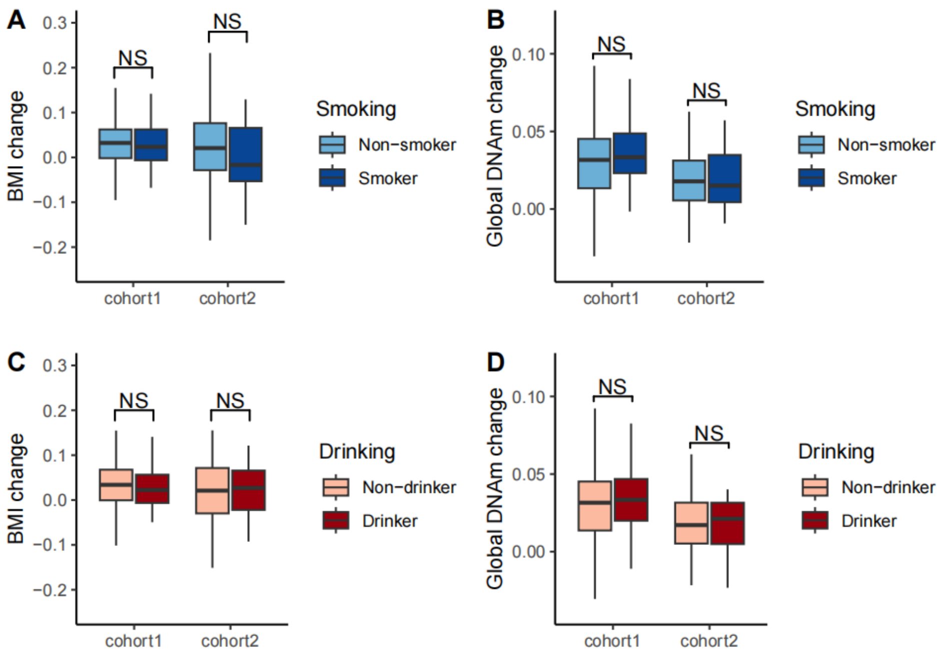

Supplementary Figure 3. PheWAS results of *VPS37B*.

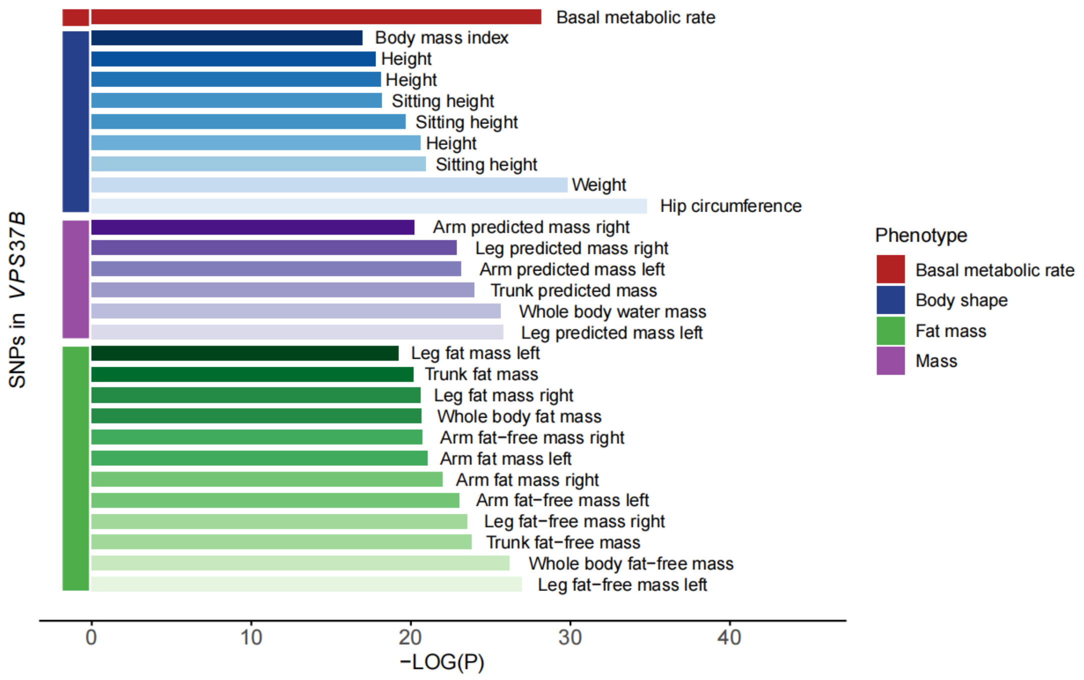

Supplementary Figure 4. The inferred regulatory process for *SLC38A4*. The eQTL variant can alter the binding affinity of transcription factors or other regulatory proteins to the enhancer region, thereby modulating its regulatory activity. Then, the active enhancer element, affected by the eQTL variant, may regulate the DNA methylation status of nearby CpG sites through its regulatory activity. Besides, a significant chromatin interaction is observed between the enhancer harboring the eQTL and the promoter region of the downstream gene *SLC38A4*. This 3D interaction brings the enhancer and the gene into close spatial proximity within the three-dimensional chromatin architecture of the nucleus. In summary, the regulatory process involves the interplay between genetic variation, enhancer activity, chromatin interactions, and gene expression, ultimately influencing the transcriptional regulation of *SLC38A4* and potentially modulating cellular function and metabolism.

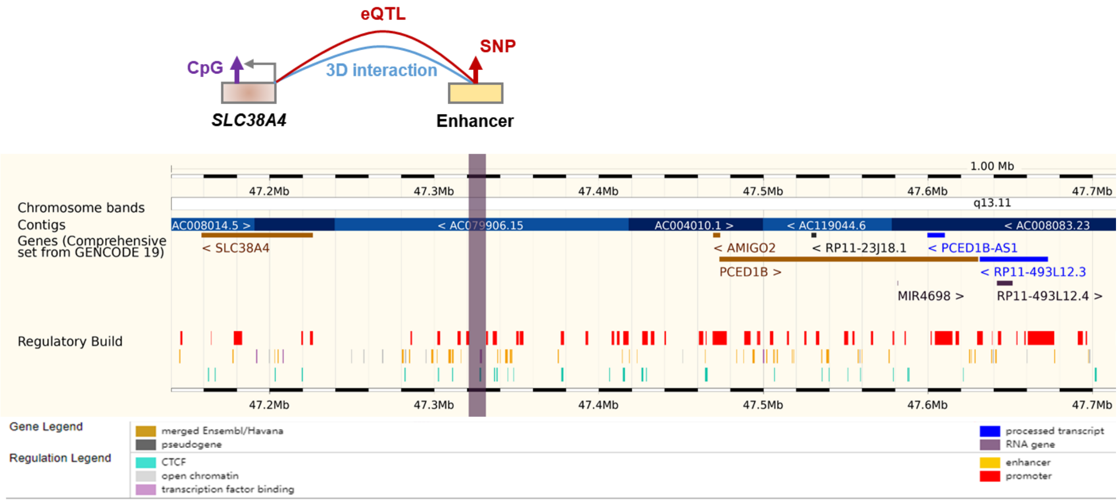

Supplementary Figure 5. Regulatory activity of cg10848724. (A) The activity of the genomic region where cg10848724 locate. From top to bottom, the bars show the location, GC percent, Jaspar TFs, and different histone marks. The height in each bar represents the activity of the corresponding position. cg10848724 was shadowed in yellow. (B) The expression of *VPS37B* in different tissues. (C) The 3D interactions in the locus where cg10848724 locate. cg10848724 was marked in red.

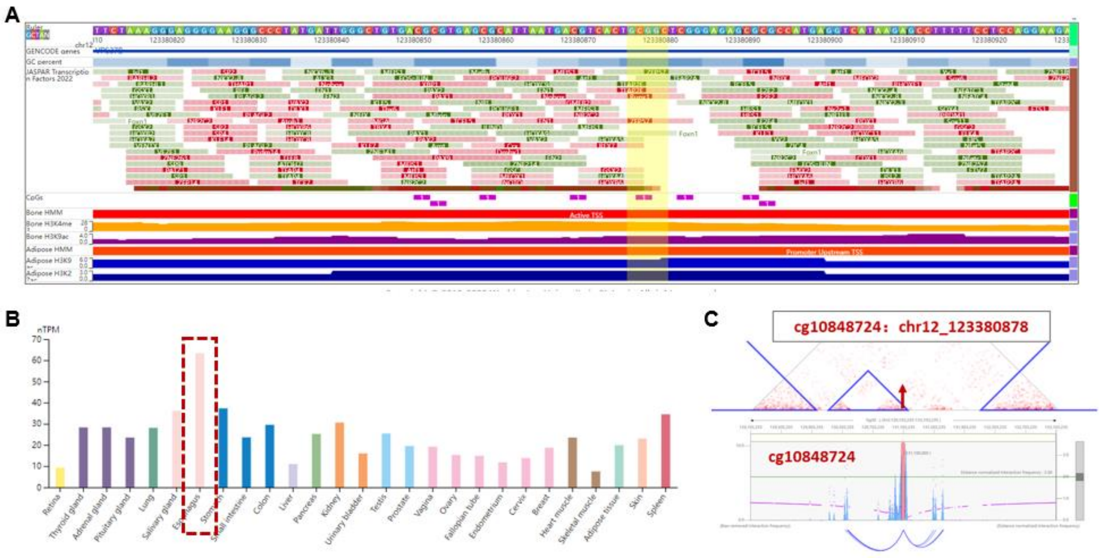

Supplementary Figure 6. Regulatory activity of cg25540824. (A) The activity of the genomic region where cg25540824 locate. From top to bottom, the bars show the location, GC percent, Jaspar TFs, and different histone marks. The height in each bar represents the activity of the corresponding position. cg25540824 was shadowed in yellow. (B) The expression of *TRIM15* in different tissues. (C) The 3D interactions in the locus where cg25540824 locate. cg25540824 was marked in red.

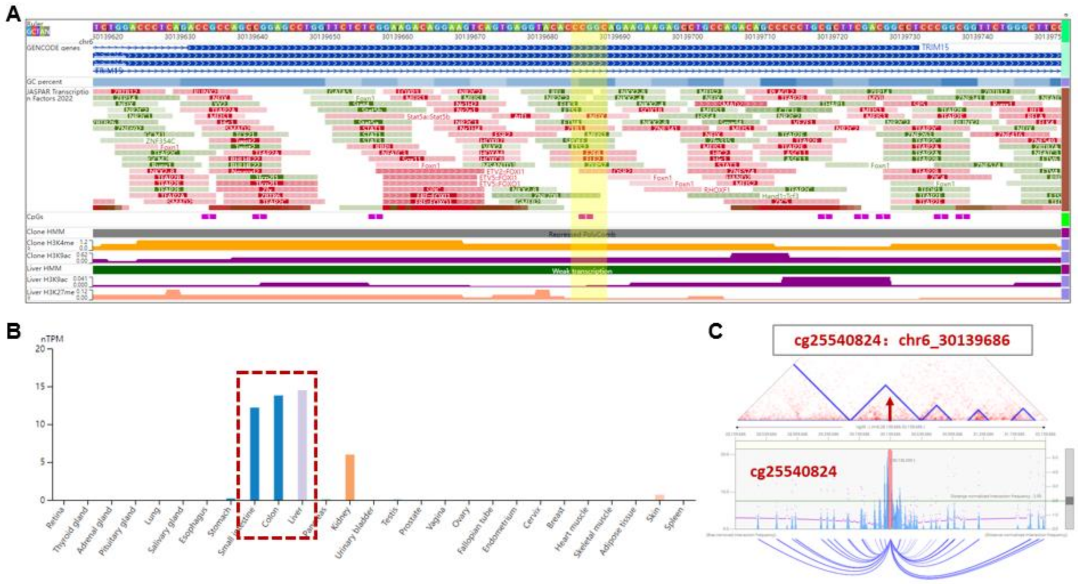

Supplementary Figure 7. Functional enrichment analysis. (A) GO enrichment of genes where the 20 most significant CpGs locate. (B) TF enrichment of the annotated genes. (C-D) Pathway enrichment of the annotated genes in Reactome pathways (C) and KEGG pathways (D).

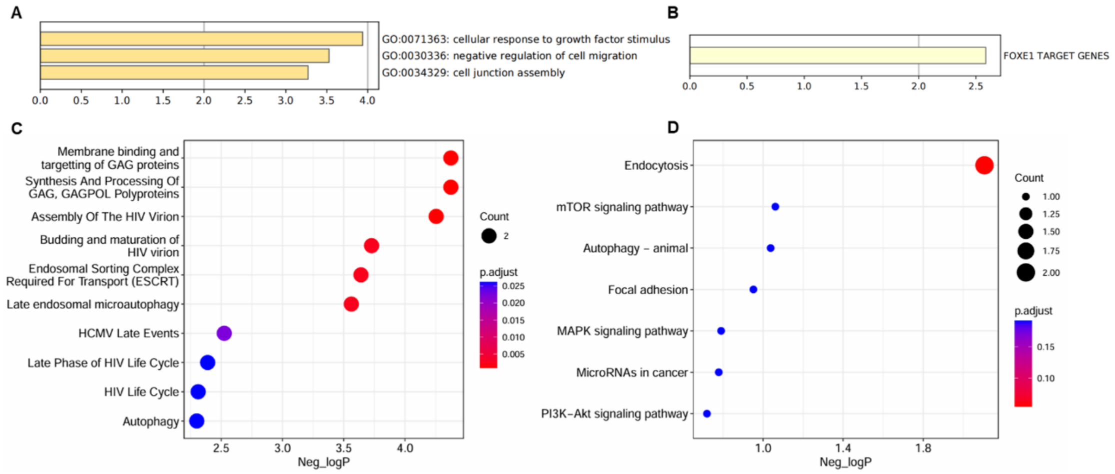

Supplementary Figure 8. Sex stratification analysis. (A-B) Volcano plot of the EWAS results in males (A) and females (B). The blue and red dots represent CpGs that passed the threshold of P < 0.05 and log2FC > 1 respectively in males and females. (C) QQ plot of effect sizes of EWAS results in males and females.

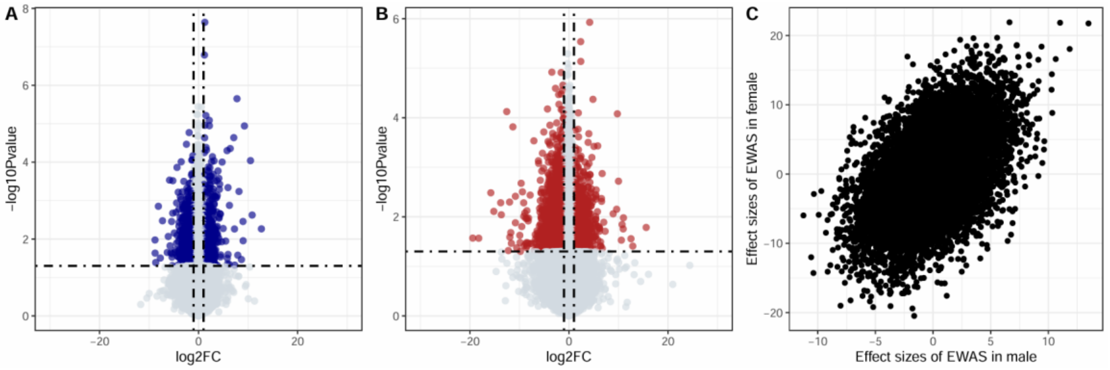

Supplementary Figure 9. Comparison of EWAS significance between three published BMI-related CpGs and randomly selected CpGs, in terms of cross-sectional EWAS of baseline BMI (A-C), cross-sectional EWAS of follow-up BMI (D-F), and longitudinal EWAS of BMI change (G-I). *** represents t-test $P < 0.005$; NS represents t-test $P > 0.05$.

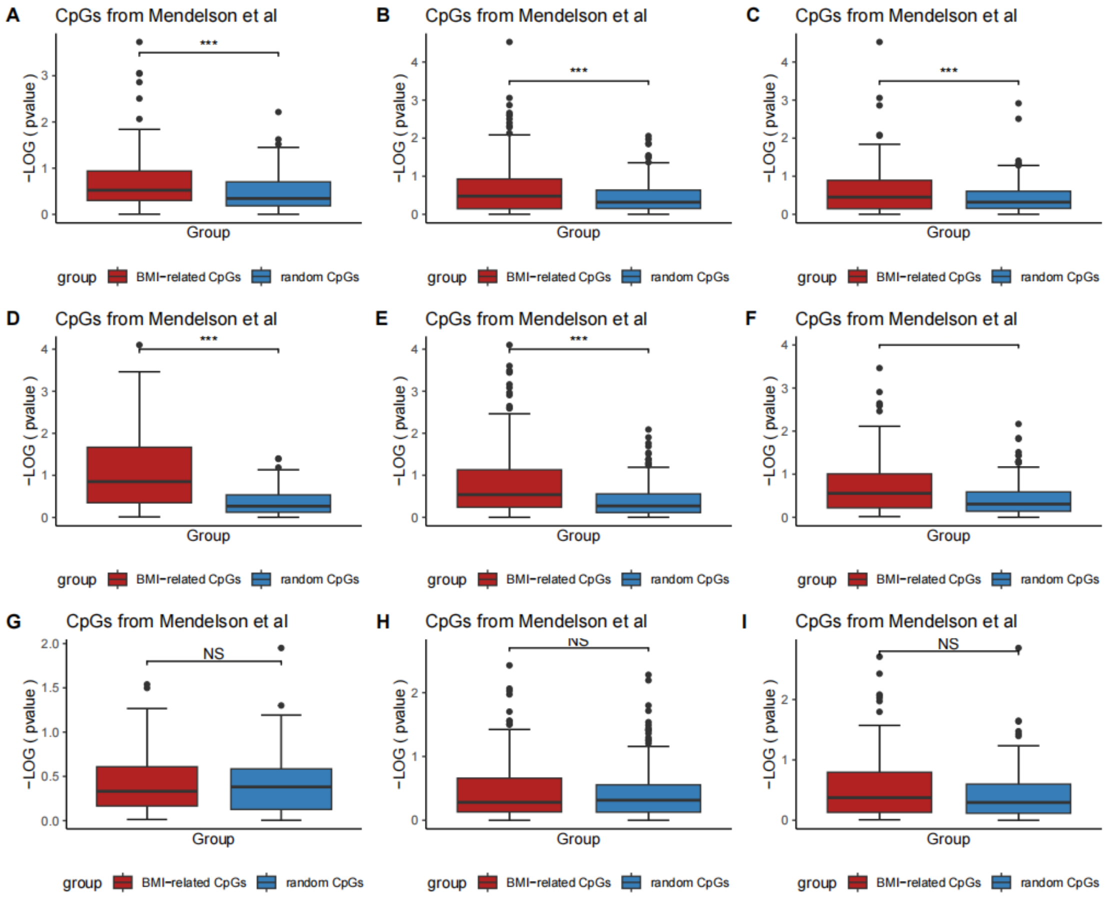

Supplementary Figure 10. The correlation between cross-sectional BMI and the methylations of the CpG identified by the longitudinal EWAS. (A-C) Correlation between baseline BMI and baseline DNA methylation. (D-F) Correlation between follow-up BMI and follow-up DNA methylation.

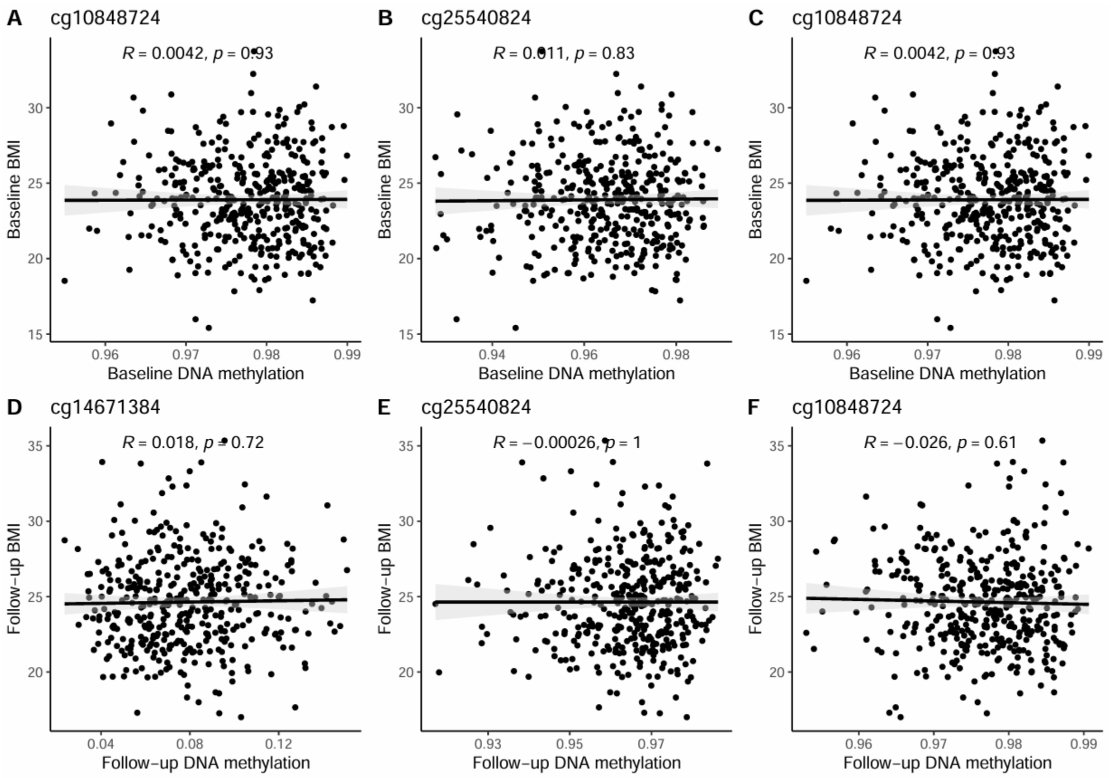

Supplementary Figure 11. Comparison of EWAS results calculated using linear mixed model (LMM) with the EWAS results of cross-sectional analysis in baseline (A) and follow-up (B). PCC: Pearson's correlation coefficient. *P* values were calculated using two-sided t-test.

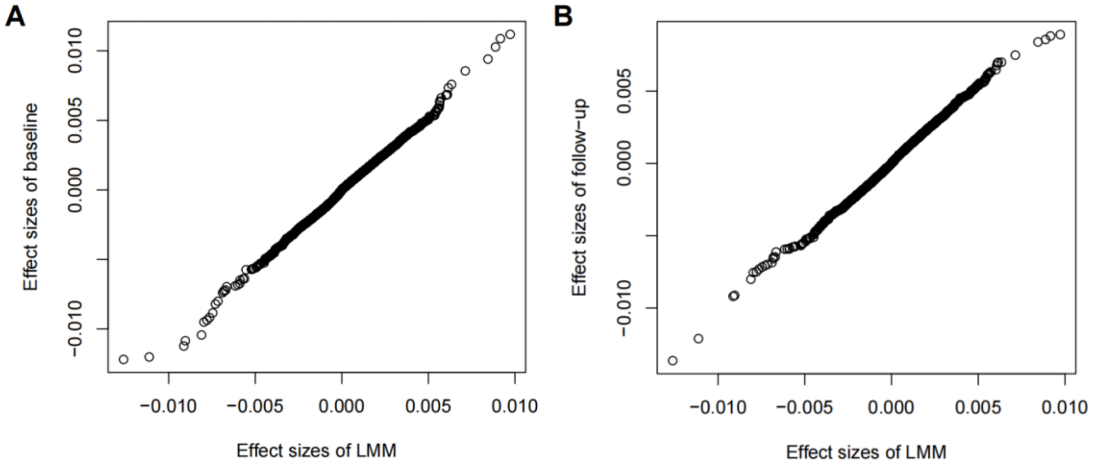

References

1. Bates D, Maechler M, Bolker B, Walker S, Christensen RHB, Singmann H, Dai B, Grothendieck G, Green P, Bolker MB: **Package 'lme4'**. *convergence* 2015, **12**(1):2.
2. Peters Timothy J, Buckley Michael J, Chen Y, Smyth Gordon K, Goodnow Christopher C, Clark Susan J: **Calling differentially methylated regions from whole genome bisulphite sequencing with DMRcate**. *Nucleic Acids Research* 2021, **49**(19):e109-e109.
3. Crocker KC, Domingo-Relloso A, Haack K, Fretts AM, Tang W-Y, Herreros M, Tellez-Plaza M, Daniele Fallin M, Cole SA, Navas-Acien A: **DNA methylation and adiposity phenotypes: an epigenome-wide association study among adults in the Strong Heart Study**. *International Journal of Obesity* 2020, **44**(11):2313-2322.
4. Cai X, Li K, Meng X, Song Q, Shi S, Li W, Niu Y, Jin L, Kan H, Wang S: **Epigenome-wide association study on short-, intermediate-and long-term ozone exposure in Han Chinese, the NSPT study**. *Journal of Hazardous Materials* 2024, **463**:132780.
5. Mendes de Oliveira E, Keogh JM, Talbot F, Henning E, Ahmed R, Perdikari A, Bounds R, Wasiluk N, Ayinampudi V, Barroso I *et al*: **Obesity-Associated GNAS Mutations and the Melanocortin Pathway**. *N Engl J Med* 2021, **385**(17):1581-1592.
6. Demerath EW, Guan W, Grove ML, Aslibekyan S, Mendelson M, Zhou Y-H, Hedman ÅK, Sandling JK, Li L-A, Irvin MR *et al*: **Epigenome-wide association study (EWAS) of BMI, BMI change and waist circumference in African American adults identifies multiple replicated loci**. *Human Molecular Genetics* 2015, **24**(15):4464-4479.
7. Kempf E, Vogel M, Vogel T, Kratzsch J, Landgraf K, Kühnapfel A, Gausche R, Gräfe D, Sergeyev E, Pfäffle R: **Dynamic alterations in linear growth and endocrine parameters in children with obesity and height reference values**. *EClinicalMedicine* 2021, **37**:100977.
8. Lee M-J: **Transforming growth factor beta superfamily regulation of adipose tissue biology in obesity**. *Biochimica et Biophysica Acta (BBA)-Molecular Basis of Disease* 2018, **1864**(4):1160-1171.
